# Experimental Evaluation and Optimisation of Sealed‐Tube Combustion Methods for Silicate Nitrogen Extraction

**DOI:** 10.1002/rcm.70193

**Published:** 2026-10-14

**Authors:** Fawn S. M. Holland, Sami Mikhail, Kirsten W. Y. Wong, Abu Saeed Baidya, Eva E. Stüeken

**Affiliations:** ^1^ School of Earth and Environmental Sciences University of St Andrews Fife UK

## Abstract

**Rationale:**

Nitrogen (N) abundance and isotopic compositions in silicate rocks are commonly measured by sealed‐tube continuous‐flow isotope ratio mass spectrometry (CF‐IRMS), but combustion protocols vary widely. It remains uncertain how the quality of data obtained from different published methods compares for differing sample types.

**Methods:**

To address this uncertainty, nitrogen abundance ([N]) and isotopic composition (δ^15^N) were measured in granite, plagioclase, alkali feldspar and biotite using tube‐cracker CF‐IRMS with seven different combustion protocols: One followed a combined tube‐ and box‐furnace method, whereas the other six used a simplified box‐furnace‐only approach, generally involving longer heating durations at lower temperatures.

**Results:**

For granite, plagioclase, alkali feldspar and biotite, we successfully identified a method that does not require a 1200°C heating step in the tube furnace without negatively impacting N yield. For granitic rocks, in cases where the highest yield methods are unsuitable (i.e., due to low [N], high sample mass requirements or lower sample melting point), we propose a suitable alternative that requires corrections on the final measured value to account for incomplete N extraction.

**Conclusions:**

For sealed tube combustion paired with continuous‐flow isotope ratio mass spectrometry, lower combustion temperatures typically reduce N extraction from silicate materials. However, increasing the combustion duration at temperatures equal to or below 1000°C can reduce, and in some cases eliminate, this yield difference.

## 1 Introduction

Nitrogen abundance ([N]) and isotopic composition (δ^15^N) in both silicate rocks and minerals can provide insight into Earth's geological nitrogen cycle [1]. However, nitrogen commonly occurs in low concentrations (typically μg/g level) in metamorphic and igneous rocks, making accurate quantitative measurements challenging.

Analytical methods to measure N in silicate geologic materials vary across studies, including neutron activation analysis (e.g., Feng et al. [2]); colourimetry (e.g., Hall et al. [3]; Feng et al. [2]); fluorometry (e.g., Feng et al. [2]); elemental analyser isotope ratio mass spectrometry (EA‐IRMS; e.g., Feng et al. [2], Boocock et al. [4]); and sealed‐tube combustion paired with continuous‐flow isotope ratio mass spectrometry (CF‐IRMS) (e.g., Boyd and Pillinger [5]; Feng et al. [2], Boocock et al. [4], Li et al. [6], Holland et al. [7]). Of these methods, the latter is perhaps the most effective approach for extracting and measuring silicate‐bound nitrogen [2, 4, 8].

For continuous‐flow isotope ratio mass spectrometry (CF‐IRMS), a sample must first be sealed in a vacuum and heated with reagents (sealed tube combustion) to liberate the silicate‐bound nitrogen and convert it into N_2_(g), which is measurable with a gas‐source mass spectrometer. This heating step varies across studies by temperature and duration and by material, generally following the trend of a high‐temperature step (≥ 950°C) to liberate the nitrogen from the crystal lattice and a lower temperature step (600°C) to convert the newly liberated nitrogen species to N_2_ (g). Busigny et al. [8] heated tubes at 950°C (6 h), followed by 600°C (2 h) for metasediments, metagabbros, altered basalts and metaperidotites. Feng et al. [2] heated tubes at 1000°C (> 3 h) and then cooled slowly to room temperature for geologic reference materials of basalt and schist, omitting the intermediate temperature step. Boocock et al. [4] heated tubes at 950°C (4 h), followed by 600°C (2 h) for basalt, granite, plagioclase, alkali feldspar, biotite, trachyte, serpentinite, shale, schist and phlogopite. Mikhail et al. [9] heated tubes to 1050°C (4 h), followed by 600°C (2 h) for strongly peraluminous granites. Li et al. [10] heated samples at 1000°C (8 h), followed by 600°C (2 h). Holland et al. [7] heated tubes to 950°C (24 h), followed by 600°C (2 h) for strongly peraluminous granites. Bustos‐Moreno et al. [11] heated tubes to 1050°C (3.5 h) for pelites, greenschists, pegmatites and metapelitic migmatites, again omitting the lower temperature heating step.

The above studies all used a box furnace for the combustion step and did not change the combustion temperature or duration for different geological materials. In contrast, Li et al. [6] compared a series of combustion variations to identify the best combustion method for a range of geological materials, including granite, plagioclase, alkali feldspar, biotite, muscovite, altered gabbro, fresh basalt, altered basalt, serpentinite, tourmaline, cordierite, clay, clinopyroxene, blueschist and eclogite. They found that although some materials have the best N extraction using lower temperatures in a box furnace (e.g., cordierite, tourmaline and clay), other materials require combustion at higher temperatures in a tube furnace before the lower temperature box furnace combustion step (e.g., granite, plagioclase, alkali feldspar and biotite) for optimal N extraction. Their 1200°C step was conducted in a tube furnace to keep the Cu_x_O_x_ reagent (separated from the sample in the sealed tube by silica wool, Figure 1) out of the hot zone to prevent it from melting and reacting through the silica glass.

**FIGURE 1:**
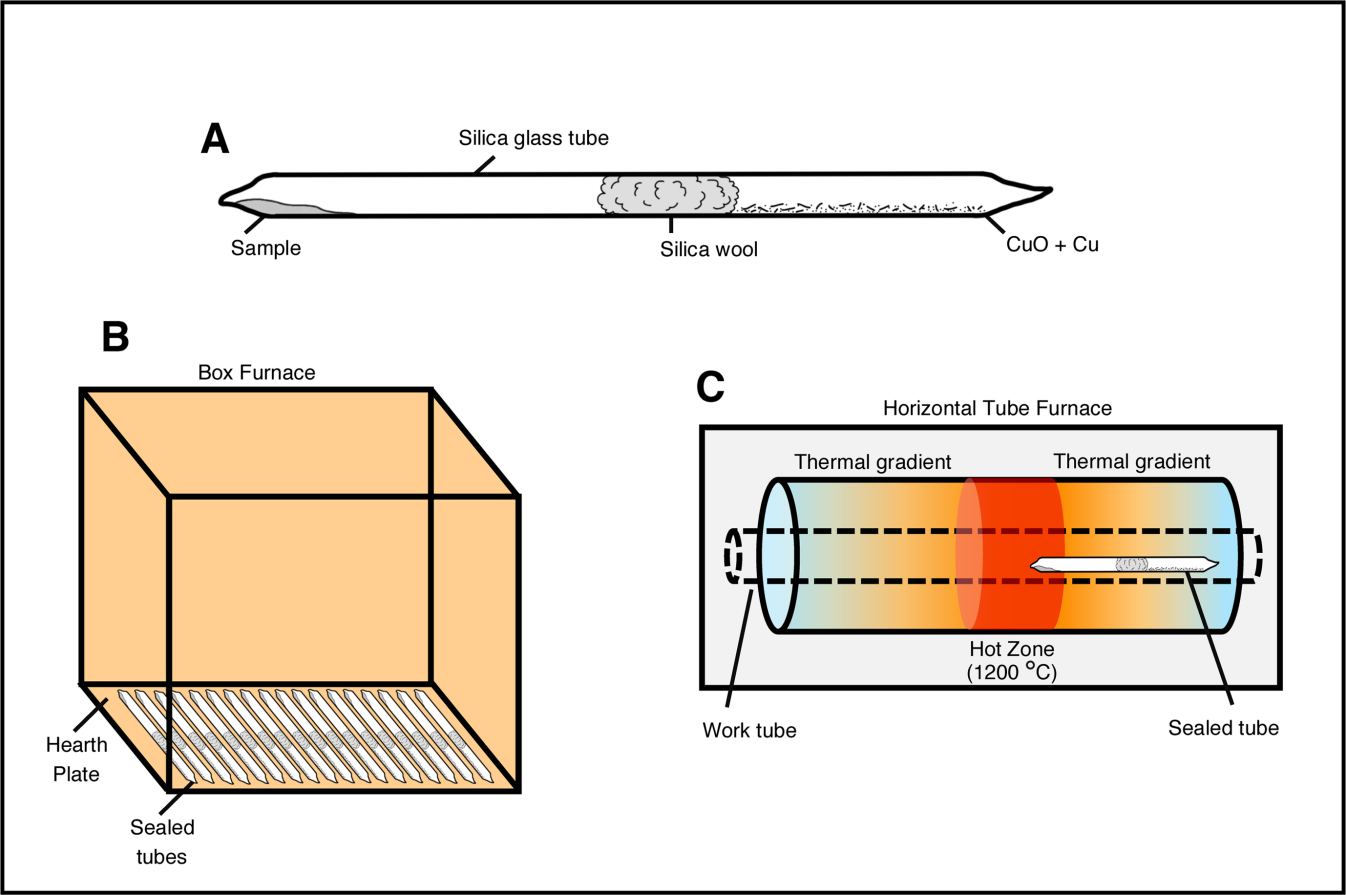
(A) Sealed tube with sample powder and CuO + Cu reagents separated by silica wool. (B) Box furnace with sealed tubes inside; all heated to the same temperature. (C) Tube furnace with a sealed tube inside; the sample end gets heated to 1200°C, whereas the CuO + Cu end stays cooler. Combustion Variation 1 needs both furnaces. Variations 2–7 only need the box furnace.

Li et al. [6] recommend the following combustion methods: for granite and plagioclase: 1200°C (60 min) in a tube furnace, followed by 900°C (8 h) and 600°C (2 h) in a box furnace; for biotite and alkali feldspar: 1200°C (30 min) in a tube furnace, followed by 900°C (8 h) and 600°C (2 h) in a box furnace. For granite, plagioclase, alkali feldspar and biotite, the highest yielding combustion method that did not require a tube furnace was 1000°C (8 h) then 600°C (2 h). However, this was the only nontube furnace method tested for plagioclase and alkali feldspar. For granite, nitrogen yields from this variation were comparable to those obtained at 900°C (8 h) then 600°C (2 h), whereas in biotite, the 1000°C variation did produce higher yields than the corresponding 900°C treatment.

Compared to their recommended combustion methods, the N yields—when baked in a box furnace at 1000°C (8 h) followed by 600°C (2 h)—are lower by 8% for granite, 44% for plagioclase, 49% for alkali feldspar and 19% for biotite (see the Supporting Information).

Compared to their recommended combustion methods, the N yields—when baked in a box furnace at 900°C (8 h) then 600°C (2 h)—are lower by 9% for granite and 35% for biotite. K‐feldspar and plagioclase were not tested at 900°C (8 h), followed by 600°C (2 h) (see the Supporting Information).

The study by Li et al. [6], although thorough, did not (and reasonably could not) test every possible combination of temperature and duration. At the University of St Andrews, we use a box furnace combustion method for granites as described in Holland et al. [7], where sample tubes are heated to 950°C (24 h), followed by 600°C (2 h), avoiding the need for a tube furnace. The approach of Li et al. [6] did not test this combustion temperature and duration, meaning that we cannot yet directly compare the effectiveness of our methods to their recommended combustion method.

Busigny et al. [8] suggested that prolonged combustion at a lower temperature (950°C for 6 h) can substitute for a short high‐temperature step (1200°C for 30 min) and showed that if the duration at the lower temperature is extended sufficiently, equivalent N extraction could be reached. However, because Li et al. [6] showed that materials respond differently to high‐temperature (1200°C) combustion, the relative performance of analytical methods should be reassessed to evaluate the accuracy of techniques used in the literature.

In this study, we evaluate seven combustion techniques applied to granite, plagioclase, alkali feldspar and biotite to determine: (1) the most effective combustion method and (2) the performance difference between the best and second‐best approach, particularly where the top‐performing approach relies on a tube furnace. If a compromise can be identified that optimises N yield and isotopic reproducibility while avoiding the need for a tube furnace, it would further simplify sample preparation, especially in cases where a tube furnace may not be easily accessible to researchers (or where larger volumes of sample are required).

An additional motivation of this study concerns the aspect of sample loss due to melting. At 1200°C, the sample powder is prone to melting (for most compositions), and the Cu_x_O_x_ reagent is more likely to react with the silica tube (inducing melting). Although melting alone is not necessarily a problem, if melting is significant enough, the melt can react with the silica glass tube and break the vacuum seal. To help avoid this, if a tube‐furnace is used, the sample can be separated from the Cu_x_O_x_ with silica wool, as shown by Li et al. [6] and illustrated in this study (Figure 1a). The tube furnace has a temperature gradient and keeps the Cu_x_O_x_ cooler, preventing melting. This does not, however, remove the risk of the sample itself melting through the glass tube. In this study, we therefore also monitored conditions where sample melting can be avoided without compromising data quality.

## 2 Materials and Methods

Nitrogen abundances ([N]) and stable isotope values (δ^15^N) were determined using the sealed tube combustion and tube‐cracker continuous‐flow isotope ratio mass spectrometry method (TC‐CF‐IRMS) at the University of St Andrews.

### 2.1 Materials

Here, we analysed four geological materials (granite, plagioclase, alkali‐feldspar and biotite) across seven different samples (Granite GR8, Granite MOB‐1, Granite 09Al105, Granite MA‐N, Albite Al‐I, Alkali feldspar FK‐N and Biotite Mica‐Fe). GR8, MOB‐1 and 09AL105 are in‐house granite samples. MA‐N, AL‐I, FK‐N and Mica‐Fe are commercially available CRPG geostandards (Table 1).

**TABLE 1:** Summary of samples selected for this study. ASI = molar Al_2_O_3_/(CaO + Na_2_O + K_2_O). Note that there is no given age for the CRPG geostandard MA‐N; however, it is from the Beauvoir granite, French Massif Central [12]. The Beauvoir granite has been dated as 315 Ma from magmatic apatite [13].

Material	Granite (MA‐N)	Granite (GR8)	Granite (MOB‐1)	Granite (09AL105)	Plagioclase (AL‐I)	Alkali feldspar (FK‐N)	Biotite (Mica‐Fe)
Sample description	CRPG geostandard: Granite	Gnt + two‐mica leucogranite	Two‐mica granite	Two‐mica granite	GRPG geostandard: Albite	CRPG geostandard: alkali feldspar	GRPG geostandard: biotite
Location	Beauvoir granite (French Massif Central)	Zanskar, Himalayas	Wyoming, United States	Niamumaodao pluton, Alxa block, North China Craton	Unknown	Madras area (India)	From a two mica granite, Massif of Saint‐Sylvestre (Central French Massif)
Age (Ma)	~315	21	2547	929	Unknown	512 ± 30	287 ± 55
ASI value	1.25	1.24	1.06	1.47	1.01	1.02	1.81
Si0_2_ (wt %)	66.6	73.66	76.76	80.04	69.49	65.02	34.4
Ti0_2_ (wt %)	0.01	0.06	0.09	0.26	< 0.005	0.02	2.5
Al_2_0_3_ (wt %)	17.62	15.23	12.2	11.3	18.57	18.61	19.5
Fe_2_0_3_ (wt %)	0.13	—	—	—	—	0.02	4.64
FeO (wt %)	0.31	—	—	—	—	0.06	18.91
T‐Fe0 (wt %)	—	0.69	0.47	—	—	—	—
T‐Fe_2_0_3_ (wt %)	0.47	—	—	2.02	0.08	0.09	25.65
MnO (wt %)	0.04	0.02	0	0.04	0.003	0.005	0.35
MgO (wt %)	0.04	0.02	0.07	1.16	0.036	0.01	4.55
CaO (wt %)	0.59	0.47	0.2	0.43	0.37	0.11	0.43
Na_2_O (wt %)	5.84	4.15	2.55	3.2	10.65	2.58	0.3
K_2_O (wt %)	3.18	4.23	6.44	1.51	0.14	12.81	8.75
P_2_0_5_ (wt %)	1.39	0.17	0.06	0.1	0.035	0.024	0.45
H_2_O^+^ (wt %)	1.08	—	—	—	0.25	0.32	2.91
H_2_O^−^ (wt %)	0.21	—	—	—	0.06	0.14	0.43
CO_2_ (wt %)	0.13	—	—	—	—	0.09	0.19
LOI (wt %)	1.82	1.11	0.46	1.41	—	0.6	2
Location Reference	[12]	[14]	[15]	[16]	NA	[17]	[18]
Age reference	See caption	[14]	[15]	[16]	NA	[19]	[19]
Major element reference	[20]	[14]	[15]	[16]	[21]	[20]	[20]

Silica glass tubes (ID = 7 mm, OD = 9.5 mm OD, initially 60 cm length) were acquired from Technical Quartz Products (United States). Silica wool (product code 21026/50) was obtained from Pelican Scientific; Cu granules (Product Code R36050.250G) were obtained from OEA laboratories Ltd.; and CuO wires (product code B1206) were obtained from Elemental Microanalysis (United Kingdom). Helium used during the analysis was purchased from BOC (United Kingdom, grade CP). The blowtorch was operated with low‐grade propane and O_2_ gas (United Kingdom). Methanol used during sample preparation was analytical reagent grade (product code: M/4000/17) from Fisher Scientific. Purified water (18.2 MΩ·cm) was generated with a Smart2Pure system (Thermo Fisher Scientific).

### 2.2 Sample Preparation

Granite Samples MOB‐1, GR8 and 09AL105 were added to borosilicate glass centrifuge vials (precleaned at 500°C for 24 h) and mixed with methanol for 1 min and then centrifuged for 10 min at 700 rpm, and the resulting supernatant liquid was decanted. This step was repeated three more times with deionised 18.2 MΩ·cm water to rinse the methanol off the powder grains. Samples were dried for > 72 h in a 70°C oven in the same centrifuge vials while loosely covered with aluminium foil to prevent contamination. After drying, plastic lids (precleaned with methanol) were added to the vials and kept at room temperature until sampled to avoid transfer contamination into a new container. CRPS geostandards MA‐N (granite), AL‐I (plagioclase), FK‐N (alkali feldspar) and Mica‐Fe (biotite) were not cleaned to avoid introducing any modifications to the geostandards before analysis.

### 2.3 Reagent Preparation

Copper oxide (CuO) pellets and copper (Cu) granules are necessary reagents for the combustion step and thus were added to every sample before evacuation and combustion. To reduce contamination, CuO pellets were precleaned in a box furnace at 950°C for 24 h, followed by 600°C for 2 h. The 600°C step allows the Cu_2_O to reabsorb the O_2_ it lost during decomposition at the 950°C step. Cu granules were precleaned in an evacuated silica glass tube at 950°C for 24 h. The vacuum is required for this cleaning step to prevent the Cu from oxidising in atmospheric air at high temperatures.

### 2.4 Preparation of Silica Tubes and Wool

Sixty‐centimetre glass tubes were halved into 30‐cm tubes using an oxy‐propane blowtorch. Cut ends were fire‐polished using the same oxy‐propane blowtorch to round the rough‐cut edges. This helped create a better seal for the vacuum step and reduced wear and tear of the associated rubber O‐rings of the ultratorr fittings (Swagelok) on the vacuum line. These new 30‐cm silica tubes were precleaned along with silica wool in a box furnace at 1000°C for 24 h.

### 2.5 Sample Loading and Creation of Sealed Tubes

Into 30‐cm silica glass tubes, 30–400 mg of sample powder was loaded. Silica wool (10–30 mg) was added to the tubes and pushed down until about 10–15 cm away from the sample end (Figure 1a) using a methanol‐cleaned stainless‐steel spatula. To keep them physically separated from the sample powder, 1.0–1.1 g of copper oxide wire and 0.2–0.3 g of copper granules were added last, with the silica wool acting as the barrier (Figure 1a). The additional 0.2–0.3 g of Cu was added to facilitate the complete conversion of oxidised species of nitrogen to N_2_.

These loaded tubes were then attached to a vacuum line and evacuated for > 24 h down to < 9.9 × 10^−7^ mbar using a combination of a rotary pump and a turbo‐modular pump. Vacuum efficiency was supported by using a liquid nitrogen trap (Figure 2b). During this step, once samples were below 1 × 10^−4^ mbar, they were preheated at 120°C using fibreglass heating tape around metal tube heaters to remove moisture on the powder grains. Before and after preheating, sample tubes were gently tapped to liberate any trapped air in the pore spaces between powder grains. Complete evacuation of pore spaces between powder grains using this method was previously verified by Holland et al. [7], who found that increasing powder mass from zero to 0.7 g had no detectable effect on measured sample N (ug). Once samples were below 9.9 × 10^−7^ mbar, they were sealed using an oxy‐propane blowtorch to preserve the strong vacuum in each sample tube. During the sealing process, the blowtorch flame was in contact with the silica glass for 30–40 s per tube. Sealing times were kept short and consistent across all samples to ensure continuity between samples.

**FIGURE 2:**
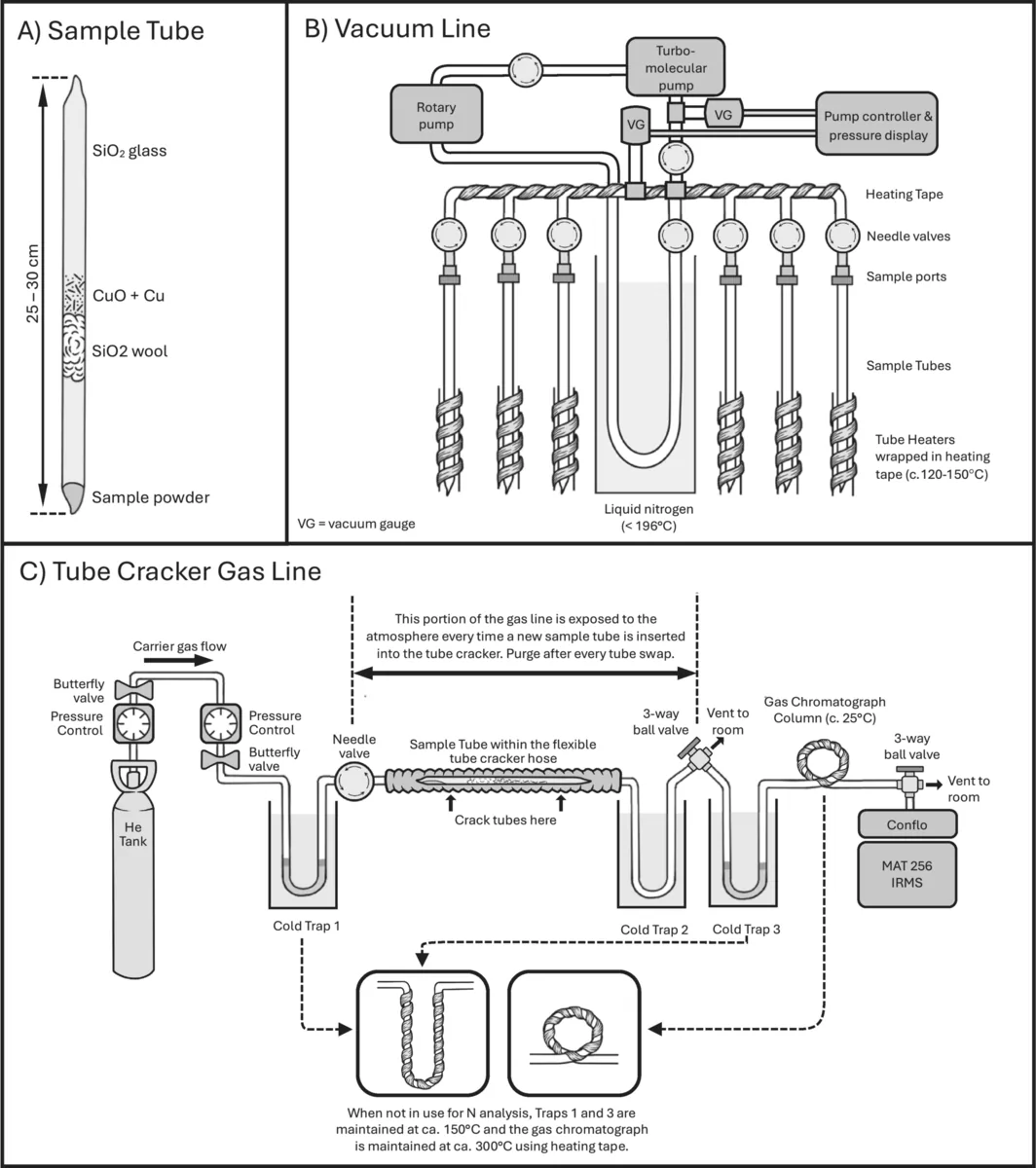
(A) Silica sample tube sealed with sample powder, silica wool, CuO wire and Cu pellets. (B) Vacuum line setup showing six sample tubes connected. Tube heaters help remove surface volatiles from the sample powder. Heating tape and liquid nitrogen, paired, create a strong temperature gradient, aiding the vacuum. (C) Tube cracker gas line showing carrier gas transporting samples (cracked in the tube cracker) to the mass spectrometer.

### 2.6 Combustion of Sealed Tubes

Seven different combustion methods were used on our samples (Table 2). Variations 2–7 were conducted using a box furnace only. Variation 1 included an extra step in a tube furnace where the sample‐end of the sealed tube was held at 1200°C for 30–60 min as recommended by Li et al. [6] and then cooled to room temperature before being placed into a box furnace for the remaining heating steps.

**TABLE 2:** Summary of combustion sequences for Variations 1–7 in this study. Only Variation 1 requires a tube furnace, as recommended for these materials by Li et al. [6]. The tube furnace is only used for the 1200°C step.

Combustion variation number	Combustion sequence	Tube furnace required?	Box furnace required?	Comment
1	1200 ° C (30–60 min), 900 ° C (8 h), 600 ° C (2 h)	Yes	Yes	Recommended by Li et al. [ 6 ] .
2	900 ° C (8 h), 600 ° C (2 h)	No	Yes	Tested by Li et al. [ 6 ] . This tests the impact of the removing the 1200°C step from Variation 1. Li et al. [ 6 ] found that removing the 1200°C step from Variation 1 (to make this Variation 2) had a negative impact on N yield for granite and biotite.
3	900 ° C (24 h), 600 ° C (2 h)	No	Yes	This tests the impact of increased time at 900°C
4	950 ° C (24 h), 600 ° C (2 h)	No	Yes	Used by Holland et al. [ 7 ] . This tests the impact of increasing temperature from 900°C to 950°C
5	1000°C (24 h), 600°C (2 h)	No	Yes	This tests the impact of increasing temperature from 950°C to 1000°C
6	950°C (48 h), 600°C (2 h)	No	Yes	This tests the impact of increased time at 950°C
7	1000°C (16 h), 600°C (2 h)	No	Yes	This tests the impact of decreased time at 1000°C

Li et al. [6] recommend the following combustion protocols for granite, plagioclase, alkali feldspar and biotite:

- Granite: 1200°C (60 min), 900°C (8 h) and 200°C (2 h).
- Plagioclase: 1200°C (60 min), 900°C (8 h), and 200°C (2 h).
- Alkali feldspar: 1200°C (30 min), 900°C (8 h), and 200°C (2 h).
- Biotite: 1200°C (30 min), 900°C (8 h), and 200°C (2 h).

When the sealed sample tube was placed in the tube furnace, the sample end was positioned fully within the hot zone, whereas the other end of the tube, containing the CuO + Cu reagents, was kept significantly cooler by the strong thermal gradient across the length of the furnace (Figure 1c). The tube furnace was heated from room temperature to 1200°C at a ramp rate of 10°C–15°C/min, kept at 1200°C for 30–60 min (as per recommendations by Li et al. [6]) and then allowed to cool to room temperature.

When the sealed tube was placed in the box furnace, the entire sealed tube was exposed to the same temperature (Figure 1b). The box furnace was heated from room temperature to 900°C, 950°C or 1000°C at a ramp rate of 15°C–20°C/min; kept at its maximum temperature for 8–48 h (depending on the combustion variation); then cooled to 600°C; kept at 600°C for 2 h; and then allowed to cool to room temperature.

For Variation 1, the 1200°C tube furnace step is to liberate NH_4_^+^ from the crystal lattices. The 900°C box furnace step causes CuO to decompose into Cu_2_O and O_2_ [22]. The O_2_ oxidises reduced forms of the liberated nitrogen species. At 600°C, the Cu and Cu_2_O reduce any nitrogen oxides into N_2_.

For Variations 2–7, the maximum temperature has the combined effect of liberating NH_4_^+^ from the crystal lattices and causing CuO to decompose into Cu_2_O and O_2._ The O_2_ still oxidises reduced forms of nitrogen species, and at 600°C, Cu and Cu_2_O again reduce any nitrogen oxides into N_2_.

### 2.7 Continuous‐Flow Isotope‐Ratio Mass Spectrometry

The sample tube was placed in a flexible metal hose (L = 41 cm, from Swagelok, United Kingdom) as part of a gas line of continuously flowing helium (45–50 mL/min) connected to an isotope‐ratio mass spectrometer (MAT 253 IRMS, Thermo Fisher Scientific) via a Conflo IV (Thermo Fisher Scientific). The gas line was purged for approximately 10 min with a gas purification trap (containing a 5 Å molecular sieve from Elemental Microanalysis, United Kingdom) under liquid nitrogen, until the background levels (m/z 28 and m/z 29) returned to baseline. A line test was conducted before sample analysis to confirm low background values. Liquid nitrogen was placed around a second gas purification trap (without a molecular sieve to capture H_2_O and CO_2_) and a final sample trap (containing the same 5 Å molecular sieve). Twelve minutes was allowed to pass while any background nitrogen from the gas line could be collected in the sample trap. The sample trap was then thawed with hot water (~55°C), and the trapped N_2_ from the sample trap was transported to the mass spectrometer (Figure 2c). Once low line blanks were confirmed, liquid nitrogen was added back around the sample trap and the sample tube in the flexible metal hose was snapped, allowing the N_2_ within to be carried to the sample trap. After 12 min, the sample trap was thawed again, and the N_2_ was carried to the mass spectrometer. The time between the line test and sample analysis is approximately 20 min. By repeated line tests, Holland et al. [7] found the line blank reduced by 17% between line test and sample analysis. Therefore, a 17% correction is applied to the line test when calculating line‐test corrections for sample analysis. Blank tube tests (containing CuO, Cu ± silica wool, but no sample powder) were also tested and subtracted from sample analysis for accurate nitrogen ([N]) and δ^15^N calculations.

### 2.8 Calibration of N and δ^15^N Measurements

Calibration lines for total nitrogen concentrations and δ^15^N values were made using United States Geological Survey standards USGS61 and USGS62 (caffeine) as well as USGS40 and USGS41a (glutamic acid). Sample MA‐N was used for quality control. Holland et al. [7] previously measured a total N content of 86.1 ± 4.4 μg/g (1 SD) and a δ^15^N value of +5.5‰ ± 0.3‰ (1 SD) with Method Variation 4. In this study, MA‐N was found to have 86.4 ± 0.9 μg/g N and a δ^15^N value of +5.4‰ ± 0.1‰ for the same combustion variation as used in Holland et al. [7] (Variation 4: furnace 950°C [24 h], 600°C [2 h]). The combustion method in this study with the highest N yield for MA‐N, however, was Variation 1, which measured 100.0 ± 0.4 μg/g and +5.8‰ ± 0.2‰. Errors reported in this study are given as ± 1 standard error (SE) to account for the varying number of replicates between materials.

### 2.9 Determining Whole Rock Mineralogy With Powder X‐Ray Diffraction (PXRD)

The mineralogy of the granite samples may have a control on the release of nitrogen under different combustion conditions. Whole‐rock granite samples were available to our lab in powdered form; hence, no petrographic analysis was possible. To determine the precise mineralogy of each of the four granite samples in this study, we used powder X‐ray diffraction (PXRD).

Powdered samples were added to 0.7‐mm glass capillary tubes (Product Code: SGCT 0.7 from Capillary Tube Supplies Ltd) and sealed with a small flame. The capillaries were rotated on a spinning stage while exposed to Mo Kα1 radiation and measured using a STOE STADI P diffractometer. PXRD analyses were done at room temperature from 2.5° to 40° (2θ) with a scan rate of 2.75° (2θ)/step in capillary Debye–Scherrer mode with acquisition times of 1 h 25 min. Raw data were converted from .raw format files to .xy format files using powDLL software [23]. The data were exported as molybdenum (Mo) 2θ data and manually converted to copper (Cu) 2θ data using Braggs law and assuming constant d‐spacing (Equation 1), where λ_Mo_ = 0.7093 Å and λ_Cu_ = 1.5406 Å [24].

(1) $$2\theta_{\mathrm{Cu}}=2*\sin^{-1}\left(\frac{\lambda_{\mathrm{Cu}}}{\lambda_{\mathrm{Mo}}}\right)\left(\sin\left(\frac{2\theta_{\mathrm{Mo}}}{2}\right)\right)$$

The resulting data were plotted as a diffractogram with 2θ_Cu_ on the *x* axis and intensity on the y‐axis. Minerals present within the spectra were identified by peak‐matching using the powdR software [25], and reference library 20260123‐newD8lib.R (S. Hillier, personal communication, 29 July 2026). Full pattern summation was run in manual mode, using the BFGS optimisation routine and Rwp objective function to minimise for best accuracy, as suggested by Butler and Hillier [25]. The internal standard used for peak alignment was Quartz (Qua11P996730). All phases selected for full pattern summation are listed in the Supporting Information.

## 3 Results

Results for measured [N] and δ^15^N values across the 7 combustion variations are plotted in Figure 3 and shown in Table 3. In the following, we will mainly focus on the comparison between Variation 1 (recommended by Li et al. [6]) and Variation 4 (equivalent to Holland et al. [7]), but we also highlight trends observed from other methods. Variation 2 is equivalent to one of the box‐furnace‐only methods tested by Li et al. [6].

**FIGURE 3:**
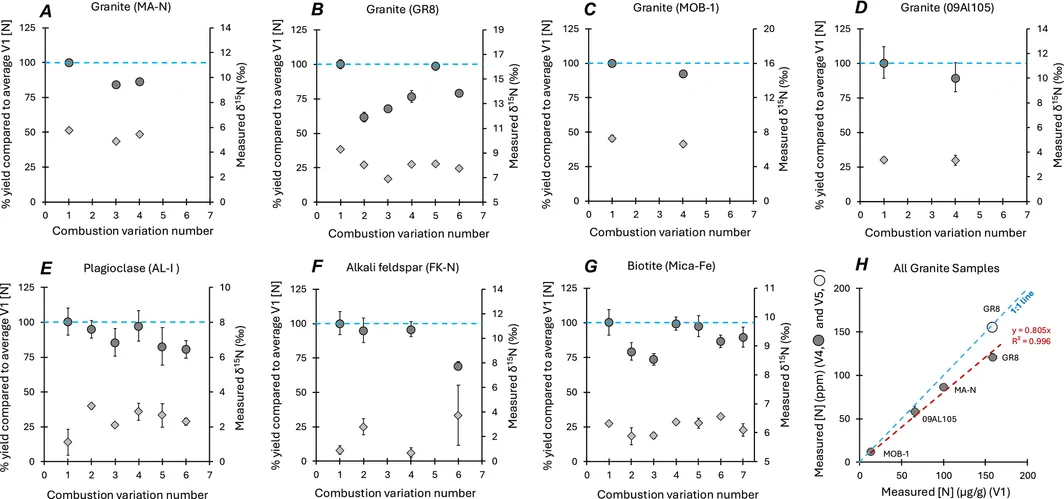
(A)–(G) show the calculated nitrogen yield from Variations 1–7 (expressed as a percentage of the average measured [N] (μg/g) for Variation 1), and the calculated δ^15^N values, for all samples in this study. Circles represent percentage [N] yield values; diamonds represent δ^15^N values. Error bars are ± 1 SE. The blue dashed line represents 100% yield compared to the nitrogen concentration recorded for Variation 1. Panel H shows measured [N] for Variations 4 and 5 (V4 and V5) plotted against variation 1 (V1) for all granite samples. The blue dashed line shows a 1:1 line. If both methods were equal in N yield, all samples would plot along this line. The red dashed line shows the linear trend line for plotted samples of V4 versus V1. No dashed line is presented for V5 versus V1 as this one data point sits on the 1:1 line. Concentrations for V4 are ~20% lower than for V1 (*y* = 0.805*x*). Concentrations for V5 are within error of V1.

**TABLE 3:** Measured [N] (μg/g) and δ^15^N (‰) in granite (MA‐N, GR8, MOB‐1, 09AL105), plagioclase (AL‐I), alkali feldspar (FK‐N) and biotite (Mica‐Fe) across 7 variations of the combustion method. Blank contribution is expressed as a percentage of the raw measured sample area (Vs) that was attributed to the blank (CuO + Cu blank, including the line blank) before blank correction. The blank‐corrected sample area (Vs) is calibrated to determine the N mass (μg), which is subsequently used to calculate [N] (μg/g). The blank‐corrected sample area (Vs), blank area (Vs) and blank δ^15^N (‰) are used to calculate the blank‐corrected measured sample δ^15^N (‰), which is then calibrated to determine the sample δ^15^N (‰). Sample data are reported as mean and standard error (SE). Individual replicate data are available in the Supporting Information.

Material	Variation Number	No. of replicates	Calculated mean [N] (μg/g)	[N] SE (μg/g)	Calculated mean δ ^15^ N (‰)	δ ^15^ N SE (‰)	Calculated mean blank contribution (%)	Blank contribution SE (%)
Granite (MA‐N)	1	3	100.0	0.4	5.8	0.2	3.0	0.1
	3	2	84.2	1.9	4.9	0.2	4.2	0.1
	4	9	86.4	0.9	5.4	0.1	3.4	0.5
Granite (GR8)	1	4	157.9	2.7	9.3	0.1	2.6	0.3
	2	4	97.3	3.8	8.0	0.0	3.2	0.2
	3	2	106.8	1.0	6.9	0.2	3.3	0.1
	4	8	120.8	4.7	8.1	0.2	2.6	0.3
	5	2	155.7	1.8	8.1	0.1	2.3	0.3
	6	2	124.7	0.1	7.7	0.0	2.1	0.0
Granite (MOB‐1)	1	2	13.2	0.1	7.3	0.4	28.1	0.1
	4	3	12.2	0.2	6.6	0.4	10.3	1.5
Granite (09AL105)	1	3	65.6	3.7	3.4	0.2	8.9	4.2
	4	5	58.6	3.5	3.3	0.4	9.6	2.2
Plagioclase (albite AL‐I)	1	5	10.4	0.5	1.1	0.7	29.1	1.5
	2	4	9.8	0.2	3.2	0.2	22.1	3.0
	3	2	8.8	0.6	2.1	0.1	17.0	2.8
	4	5	10.1	0.7	2.9	0.5	18.3	4.8
	5	2	8.5	1.0	2.7	0.7	35.5	7.3
	6	2	8.4	0.3	2.3	0.2	22.8	2.0
Alkali feldspar (feldspath FK‐N)	1	4	9.6	0.4	0.9	0.3	29.6	5.6
	2	2	9.1	0.5	2.8	0.7	29.9	0.5
	4	3	9.2	0.1	0.7	0.4	14.7	0.7
	6	3	6.6	0.0	3.7	2.4	52.1	1.3
Biotite (mica‐Fe)	1	2	193.0	8.9	6.3	0.0	4.1	0.3
	2	2	152.4	5.1	5.9	0.3	2.2	0.4
	3	2	142.0	1.5	5.9	0.1	2.9	0.4
	4	2	191.5	0.4	6.4	0.0	2.6	0.6
	5	1	187.7	6.2	6.3	0.2	3.2	N/A
	6	2	166.9	1.0	6.6	0.0	1.9	0.4
	7	3	172.6	5.9	6.1	0.2	4.2	0.4

### 3.1 Granite

Four granite samples were tested: MA‐N, GR8, MOB‐1 and 09AL105. When Combustion Variation 4 was used, the N yield was 77%–92% compared to variation 1 (Figure 3). Variation 5 (1000°C for 24 h, followed by 600°C for 2 h) is equivalent to Variation 1 for N yield; however, some granite samples melted through the tube at this temperature, as seen in MA‐N. Substantial melting can break the vacuum seal and cause sample gas loss. When comparing Variation 4 to Variation 1, the variability in offset between the four granite samples does not strongly correlate with major element geochemistry or mineralogy including: aluminium saturation index (ASI) (*R* ^2^ = 0.0), Al_2_O_3_ (*R* ^2^ = 0.3), CaO (*R* ^2^ = 0.3), Na_2_O (*R* ^2^ = 0.2), K_2_O (R^2^ = 0.0), plagioclase (*R* ^2^ = 0.1), alkali‐feldspar (*R* ^2^ = 0.0) and biotite (R^2^ = 0.4) (Figure 4 and the Supporting Information).

**FIGURE 4:**
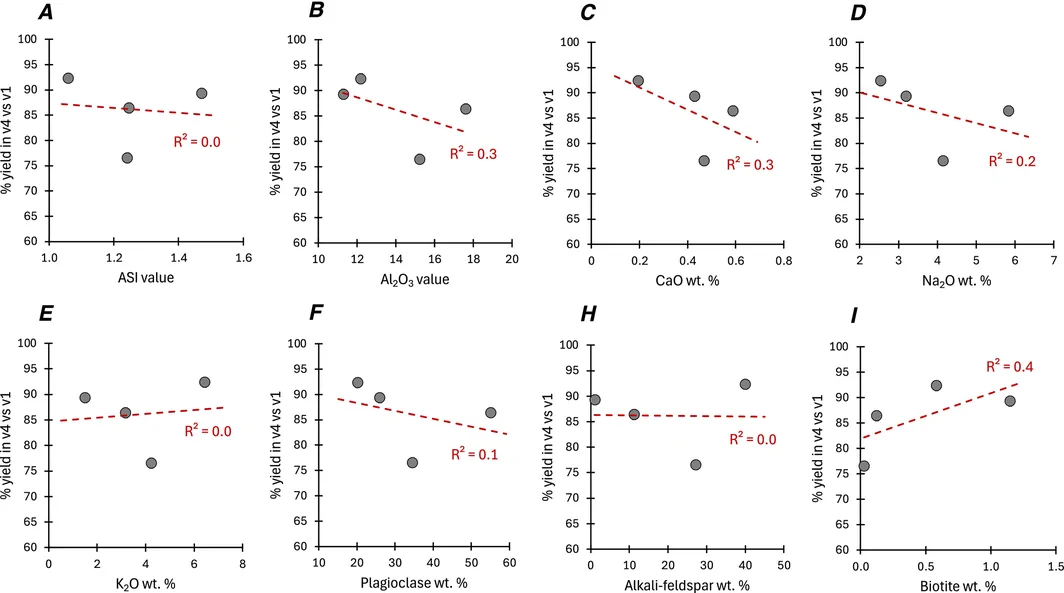
Calculated nitrogen yield from variation 4 (v1) (expressed as a percentage of the average measured [N] [μg/g] for Variation 1 [v1]) plotted against ASI value, Al2O3, CaO, Na2O, K2O, plagioclase, alkali‐feldspar and biotite.

### 3.2 Plagioclase

One sample of plagioclase was tested: AL‐I. When Combustion Variation 4 was used, the N yield was within error of Variation 1. Variation 2 is the second‐best method for N yield, at 95% yield compared to Variation 1, and still within error. The remaining variations resulted in 80%–85% yield compared to Variation 1.

### 3.3 Alkali Feldspar

Variations 1, 2 and 4 are all within error for N yield. These are equivalent methods. Variation 6 had a significantly lower yield on average (69% compared to Variation 1).

### 3.4 Biotite

Variations 1, 4 and 5 are all within error for N yield. The remaining variations resulted in 74%–89% yield compared to Variation 1.

### 3.5 Sample Melting

At 1200°C, all samples underwent a degree of melting and/or annealing as evidenced by the once‐loose powder now forming a vitreous cohesive mass. However, the severity of sample melting and subsequent reaction with the silica glass tube varied by sample type and sample mass. For some samples, melting was significant enough that the melt reacted with the silica glass tube and caused it to crack, losing the vacuum seal and rendering the sample immeasurable (Figure S1).

Figure 5 shows the weighed masses of samples of granite, plagioclase, alkali feldspar and biotite that were all baked at 1200°C as per Combustion Variation 1, sorted into two categories. The first category is for samples that survived the 1200°C combustion step (sample intact). The second category is for samples which melted through the glass and broke the vacuum seal (sample lost).

**FIGURE 5:**
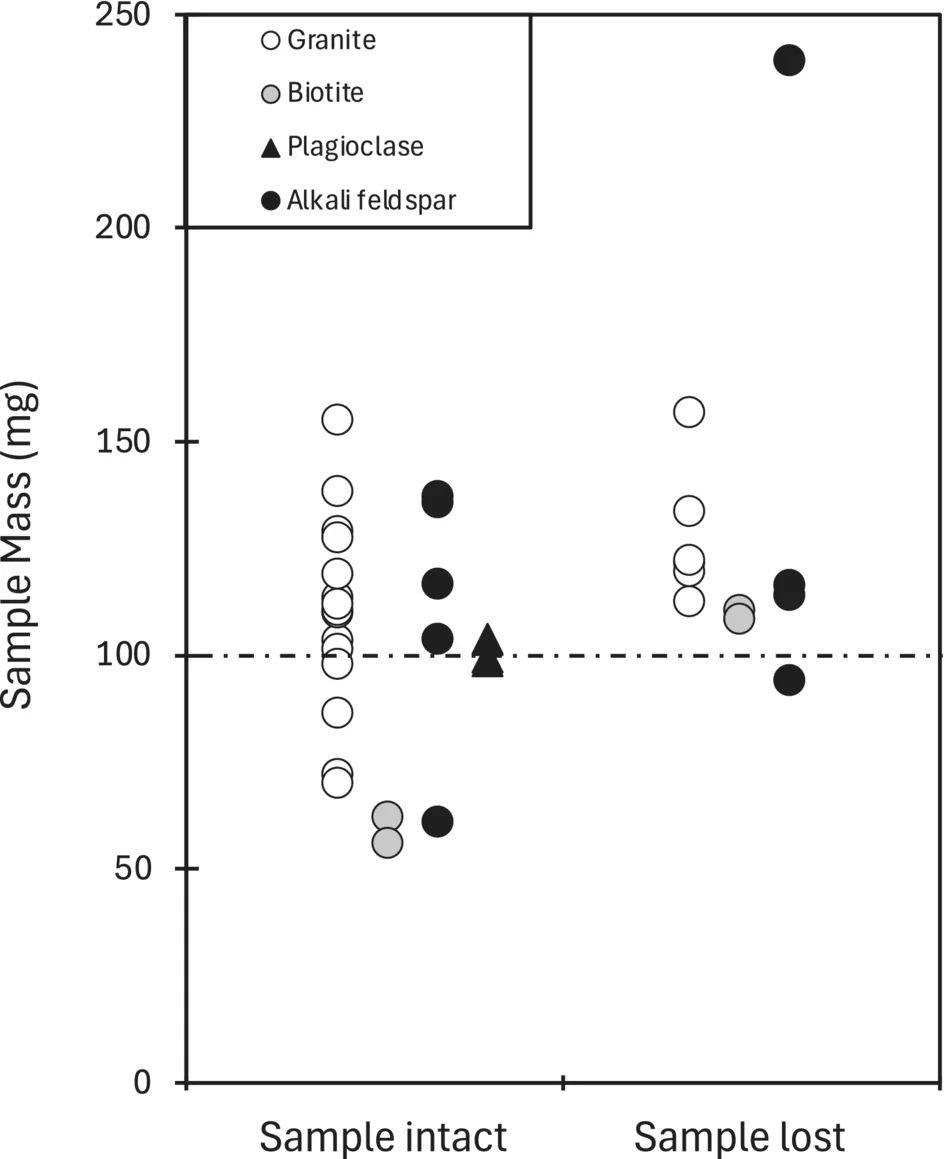
Plot shows samples of granite, plagioclase, alkali feldspar and biotite that were combusted at 1200°C as per Variation 1. Some samples survived this combustion and went on to be measured for [N] and δ^15^N (sample intact). Other samples melted and reacted with the silica glass tube, and the vacuum seal was broken, losing the sample (sample lost). The dashed line shows 100 mg; most samples lost were > 100 mg.

For granite, samples that survived 1200°C combustion were 70–155 mg; samples that did not survive this step were 113–157 mg. For plagioclase, all samples survived 1200°C combustion at 99–104 mg. For alkali feldspar, samples that survived 1200°C combustion were 61–137 mg; samples that did not survive this step were 94–239 mg. For biotite, samples that survived 1200°C combustion were 56–62 mg; samples that did not survive this step were 109–111 mg. We note significant overlap between the temperatures of the two categories for a given sample type, meaning that melting cannot easily be predicted. However, the risk of melting appears to increase with increasing sample mass and is mostly avoided below 100 mg.

For samples where the vacuum seal was lost, the CuO + Cu pellets were inspected to determine if they played a role in the melting process that triggered the observed failure in the silica glass. CuO breaks down to Cu_2_O at ~700°C–800°C [22], which melts at ~1235°C. Copper melts at ~1083°C. These melting points may be lower under vacuum. Therefore, if these pellets were close enough to the hot zone, which was kept at 1200°C, they could melt and affect the vacuum seal. Upon inspection, the CuO and Cu pellets were determined to be intact (loose, unmelted, not sintered). Therefore, the pellets did not melt nor cause the observed cracking or melting of the samples. Photographs are available in the Supporting Information.

### 3.6 Determining Whole Rock Mineralogy With X‐Ray Diffraction

Key mineral abundances of the four whole rock granite samples are shown in Table 4. Complete mineralogy results from XRD full pattern summation are in the Supporting Information.

**TABLE 4:** Calculated modal abundances of key minerals in whole‐rock granite samples from this study. Data were obtained from powder X‐ray diffraction (PXRD).

Granite sample	Quartz (%)	Plagioclase (%)	K‐feldspar (%)	Biotite (%)	Muscovite (%)
	SiO_2_	(Na, Ca)Al (Si, Al)Si_2_ O_8_	(K, Na)AlSi_3_ O_8_	K (Mg, Fe)_3_ AlSi_3_ O_10_ (OH,F)_2_	KAl_2_ (Si_3_ Al)O_10_ (OH,F)_2_
MA‐N	18	55	11	< 1%	11
GR8	29	35	27	< 1%	6
MOB‐1	34	20	40	1	1
09AL105	56	26	1	1	9

Whole‐rock mineralogy did not appear to have a measurable effect on yield when comparing Variation 4 to Variation 1. The correlation between yield (%) and modal mineralogy was weak for all minerals: quartz (*R* ^2^ = 0.1), plagioclase (*R* ^2^ = 0.1), alkali feldspar (*R* ^2^ = 0.0), biotite (*R* ^2^ = 0.4) and muscovite (*R* ^2^ = 0.0) (Figure 4 and the Supporting Information).

## 4 Discussion and Recommendations

### 4.1 Granite

Li et al. [6] found that when using variation 2 (900°C for 8 h, 600°C for 2 h) compared to variation 1 (1200°C for 30 min, 900°C for 8 h, 600°C for 2 h), the N yield was suppressed by 9%. When comparing Variations 1 and 2 in our study, our data confirm that there is a reduction in yield; however, we found that this was suppressed by 38%, suggesting that different granites will react differently to changes in temperature and duration.

We have identified one method (Variation 5), which can be used without the need for a tube furnace to yield similar [N] results; however, the recorded δ^15^N value was also 1.2‰ lower. It is unclear why these two values differ or which measurement best reflects the true value.

Variation 4 [7] is not as efficient as Variation 1 [6] for N extraction in granites, with only 77%–92% yields. Isotopic compositions for three of the four granites are similar; however, the fourth granite (GR8) has a lower measured stable isotope value for Variation 4 (+8.1‰ ± 0.2‰) compared to Variation 1 (+9.3‰ ± 0.1‰), but a similar isotopic value to Variation 5 (+8.1‰ ± 0.2‰). It is unclear why these isotopic values differ or which measurement best reflects the true value. However, the difference between these two numbers would not impact the interpretation of nitrogen isotope data in Holland et al. [7].

### 4.2 Plagioclase

When comparing Combustion Variations 1 and 2 in our study, we found that the average [N] value was suppressed by only 5%, and this was still within error of Variation 1. Variation 4 is equivalent to Variation 1 for N yield; however, δ^15^N is 1.8 ‰ higher. The cause of this isotopic difference is unknown.

### 4.3 Alkali Feldspar

Li et al. [6] found that for alkali feldspar baked at 1000°C for 8 h, followed by 600°C for 6 h (not tested in this study), the N yield is only 49% compared to variation 1 (see the Supporting Information), and the isotopic value is 1.1‰ lower. This is a significant difference that strongly advocates for the need for high‐temperature combustion in a tube furnace (i.e., Variation 1). However, in this study, we show that when the temperature is lowered even further to 950°C and extended to 24 h (Variation 4 in this study), then this N yield difference completely disappears: Measured [N] and the δ^15^N remain the same as measured in variation 1.

Variation 6 had a lower N yield and a significantly higher range in δ^15^N values. As more complete extraction is observed in variation 4 (also 950°C, but 24 h instead of 48 h), these results for Variation 6 are best explained by either: partial resorption of N due to excess time at high temperatures, or high errors from blank input (50%–55% blank, higher % blank than all the rest of the data points in this study). High blanks in the batch of alkali feldspar samples for Variation 6 were due to lower sample mass (from low sample availability) and therefore a higher relative blank contribution. This can be avoided where large amounts of rock powder are available.

### 4.4 Biotite

Li et al. [6] found that when combustion of biotite is set to 900°C for 8 h, followed by 600°C for 2 h (Variation 2 in this study), the N yield for biotite is suppressed by 35%. In our study, we agree with the reduced yield but to a lesser extent, at a yield suppression of 21%. Li et al. [6] also found that when the combustion temperature is raised to 1000°C (8 h), followed by 600°C (2 h), the N yield of biotite increases slightly, to just 19% suppression.

In our study, we find that increasing the combustion time even further to 24 h for 950°C (Variation 4) or 1000°C (Variation 5) eliminates the N suppression and produces the same isotopic values as Variation 1.

### 4.5 Different N Extraction Methods for Different Geological Materials

It is clear that different geological materials require different combustion methods for optimal N yield. Because there was no N yield suppression for biotite between Variations 1 and 4, and because of its relatively low standard error percentage compared to average concentration, we suggest that biotite is the easiest to combust for N release. Although plagioclase and alkali feldspar have equivalent average concentrations of nitrogen (within 1 standard error) between Combustion Variations 1 and 4, their low nitrogen concentrations (and thus higher blank % and relative standard error) could be hiding a small yield suppression that we cannot detect with our current analytical resolution. The lower yields in granites (when combusted as per Variation 4 as opposed to Variation 1) could be caused by N hosted in plagioclase and/or alkali feldspar. An alternative possibility is that there is another factor affecting granites, causing this difference that has not yet been identified. For example, this could include inclusions within quartz, which was not explored here. Indeed, the feldspar separates show similar yields for Variations 1 and 4; however, these have overlapping error bars and are based on very low abundances (6–12 μg/g).

For all materials, the nitrogen yield for variation 6 (950°C [48 h], followed by 600°C [2 h]) is lower than variation 1 (950°C [24 h], followed by 600°C [2 h]). This could indicate that some of the nitrogen is resorbing back into the crystal structure as the combustion time is increased.

For the alkali feldspar investigated in this study, nitrogen yield in Variations 2 and 4 is dissimilar to that of Variation 1. δ^15^N values for Variations 1 and 4 are similar; however, for variation 2, δ^15^N is elevated by +2‰ (Table 3). This likely reflects sample heterogeneity (the nugget effect).

### 4.6 Sample Melting

An effective solution to the sample reacting with the silica tube would be to host the sample within a cold pressed envelope of platinum metal. However, this would be a very expensive adaptation of the method and risk introducing additional blank nitrogen to an already low [N] sample. Instead, we highlight that the risk of a sample melting through the sealed glass tube is significantly lower when baking in a box furnace at lower temperatures. However, at lower temperatures, we risk incomplete N extraction (e.g., Li et al. [6]). Therefore, a compromise is required that optimises N extraction and reproducibility for different materials while avoiding the risk of melting.

Not enough samples were combusted at 1200°C to be sure of what causes some to melt at lower masses. We can, however, suggest that in general, if using variation 1 to heat samples to 1200°C, no more than 100 mg of sample should be added to the glass tube. This will significantly lower the risk of sample loss through melting and reacting with the glass tube to the point of breaking the vacuum seal (Figure 5). This is because the less sample powder, the less melt it can produce and therefore the less the silica tube is forced to interact with this melt. The more melt that is present over an area of quartz tubing, the more likely it will melt through the glass, or react with the glass, causing it to crack and cause the vacuum seal to break.

In summary, when we compare the method used by Holland et al. [7] (Variation 4) and all other variations (2, 3, 5–7) to the preferred method by Li et al. [6] (Variation 1), we find that our variation 4 has lower risks of sample loss by melting but higher risks of incomplete N extraction. For the latter, correction factors can be applied to account for incomplete N extraction from granite (see Section 4.8). No corrections are required for alkali‐feldspar, plagioclase, and biotite.

### 4.7 Granite Mineralogy Controls on Nitrogen Release

Results show no resolvable differences in N yield—within 1 standard error—for biotite, plagioclase and K‐feldspar when comparing Variations 1 and 4. However, whole‐rock granite samples yield up to 23% lower yield for Variation 4 compared to Variation 1. One explanation for this is that the differing mineral percentages in these granites are imposing a small difference, which is small enough that it is hidden by the error bars seen in Figure 3. To address this, we calculated the compounding effects of lower mineral N yield in Variation 4 (taking the lower end standard error on Variation 4 yield and the upper standard error on Variation 1 yield). For example, although plagioclase has overlapping error bars in concentration between combustion Variations 1 and 4 (see Table 3 and Figure 3), if we take the upper concentration of plagioclase under Variation 1 (average [N] + standard error = 10.9 μg/g) and compare this to the lower concentration of plagioclase under variation 4 (average [N] – standard error = 9.4 μg/g), the yield for Variation 4 may be as low as 86% compared to Variation 1. By doing the same to biotite and alkali feldspar, their yields could be as low as 95% and 91%, respectively. We can compound the effects of these on the whole rock granites to calculate the lower bound of yield for each granite that could be accounted for by the mineralogy. To do this, we must assume that (1) all the nitrogen in the whole‐rock granite powders is exclusively stored in the following three minerals: biotite, plagioclase or alkali feldspar; (2) that the concentrations of nitrogen in the biotite, plagioclase or alkali feldspar are roughly equal (but see Honma and Itihara [26], and Boocock et al. [27], for discussion). With these assumptions in place, we calculate that whole‐rock granite samples MA‐N, GR8, MOB‐1 and 09AL105 could be up to 11%–13% lower in yield for Variation 4 compared to Variation 1 purely based on mineralogy (see the Supporting Information for calculations). This does not fully account for the 77% yield value observed for GR8, nor the 82% value for 09Al105. Hence, an additional compounding factor appears to be hindering efficient N‐extraction from granites, which could include N‐inclusions, back‐reactions between liberated N and other compounds contained within the samples, and/or heating‐induced physical changes such as sintering within the multiphase granite assemblage that could cause reduced porosity and hinder nitrogen diffusion.

### 4.8 Recommendations

Below are recommendations on the best combustion method for each geological material: granite, plagioclase, alkali feldspar and biotite. Before applying Variation 1, it is important to determine if sample melting can be avoided by using a sufficiently low sample mass. This will depend on [N], as well as blank levels and required signal/blank ratios. An approximate estimate of [N] can be obtained at relatively low cost by EA‐IRMS. Incomplete combustion of some rock types by this technique may require a correction factor; however, it can still provide a rough estimate of [N] and thus required sample amounts for sealed‐tube combustion. If the whole‐rock [N] is known, but [N] of its mineral separates to be tested are unknown, it is viable to assume that all the nitrogen is predominantly hosted by alkali feldspar and biotite, to estimate [N] in those minerals [27]. Where [N] and the melting behaviour of a new sample are unknown, we recommend a cautious approach for samples that are difficult to replace by opting for the lower temperature, longer duration combustion variation over the high‐temperature tube furnace option in Variation 1.

#### 4.8.1 Granite

When expected [N] is > 30 μg/g N and the blanks are < 0.3 μg, the sample mass can be below 100 mg while still retaining a reasonably low blank percentage contribution (~10%). If these conditions are met, and a tube furnace is accessible, we recommend using Variation 1. Weighing < 100 mg of sample should reduce the risk of sample loss by melting through the silica glass tube.

If limited sample is available, but the melting point is high enough not to react with the silica glass at 1000°C, we recommend variation 5; this will yield similar [N] values compared to variation 1, but the δ^15^N value may be slightly lower. We found an offset of −1.2‰ for Variation 5 compared to Variation 1 in the instance of Granite sample GR8, but this may require more systematic testing with a wider range of samples. This method is a lower temperature than Variation 1 and therefore could prevent it from melting if there is insufficient sample powder to replace a lost sample.

If the predicted sample [N] or melting behaviour of samples is unknown, we recommend using Variation 4 as a safer option. A 24% [N] yield correction would have to be applied (Figure 3); however, no δ^15^N correction would be required, as three of the four granites tested showed very similar δ^15^N values compared to Variation 1.

#### 4.8.2 Plagioclase

When expected [N] is high, and the sample mass can therefore be below 100 mg while still retaining a reasonably low blank percentage contribution, we recommend using Variation 1.

Where estimated [N] is low or unknown, we recommend using Variation 4. No [N] or δ^15^N correction would be required; however, the error on the δ^15^N may be up to 1.8‰ because although we see similar [N] for Variations 1 and 4, we also see different δ^15^N values (+1.1‰ vs. +2.9‰, respectively).

#### 4.8.3 Alkali Feldspar and Biotite

Although both Variations 1 and 4 are equivalent for [N] and δ^15^N, we recommend Variation 4 as this variation is less likely to cause the sample to melt and react through the glass tube, while having no adverse effect on measured [N] or δ^15^N. No corrections would be required.

## 5 Conclusions

Our results broadly agree with Li et al. [6], where adding a short (30–60 min) 1200°C step to 900°C (8 h) + 600°C (2 h) heating sequence increases N yield. However, this 1200°C step significantly increases the risk of losing sample through melting and reaction with the silica glass tubing. As a compromise, reducing the temperature and increasing the duration of the box furnace step can reduce, and in some cases eliminate, this yield difference and avoid the risk of melting. The advantage of a longer combustion in a box furnace was previously highlighted by Busigny et al. [8]; however, here we present these data for four different geological materials: granite, plagioclase, alkali feldspar and biotite. The systematics, especially for different granite samples, suggest that correction factors can be applied to measured [N] or δ^15^N values, if required.

Although the different methods evaluated here should be considered when deciding how to proceed, we highlight the fact that the resolution required in the result must be the definitive factor. We show that there is potentially up to 24% error in the measured [N] in granites, but no clear relationship was identified that would allow this difference to be predicted. Previously published data trends would not be significantly impacted by the methodology. For example, Mikhail et al. [9] showed that strongly peraluminous granites preserve a secular increase in nitrogen and phosphorus across the Precambrian–Phanerozoic transition, and Holland et al. [7] showed that nitrogen preserved in strongly peraluminous granites records a major increase in biomass burial beginning in the late Mesoproterozoic. Both results would be resolvable using the method of Li et al. [6], Holland et al. [7] or the methods assessed in this study.

## Author Contributions

**Fawn S. M. Holland:** data curation, methodology, visualization, writing – review and editing, conceptualization, investigation, funding acquisition, writing – original draft, project administration, formal analysis, validation, supervision. **Sami Mikhail:** supervision, resources, writing – review and editing, methodology, funding acquisition, visualization, project administration. **Kirsten W. Y. Wong:** investigation, project administration, validation. **Abu Saeed Baidya:** funding acquisition, visualization, investigation, project administration. **Eva E. Stüeken:** supervision, resources, methodology, visualization, writing – review and editing, conceptualization, project administration.

## Funding

This work was supported by the Natural Environment Research Council (NE/S007431/1 and NE/Z504233/1), the Engineering and Physical Sciences Research Council (EP/V034138/1) and the HORIZON EUROPE Marie Sklodowska‐Curie Actions (EP/Y026497/1).

## Supporting information


**Figure S1:** (A) Two plagioclase samples, which melted and reacted with the silica glass tube, but the reaction was not so intense that it caused the tube to crack. These samples were measurable. (B) Plagioclase (left) and biotite (right) samples, which melted and reacted with the glass tube; the reaction was so intense that it caused the tube to crack. These samples were not measurable.

**Figure S2:** Granite (gran), alkali feldspar (ksf), and biotite (bt) samples which melted, reacted with the glass tube, and caused the tube to crack. The vacuum seal was lost, and these samples were not measurable. These tubes were photographed to demonstrate that after the 1200°C combustion step, the CuO and Cu pellets were still loose and did not melt. Therefore, CuO and Cu melting did not cause the tube to fail, and the failure was caused by a property of the sample powder itself. (A) The CuO and Cu pellets fall towards the silica wool. (B) The CuO and Cu pellets fall towards the tube end, away from the silica wool. Panels A and B together demonstrate that the pellets are still loose. (C) Close‐up of the CuO and Cu pellets. (D) Close‐up of the sample end of the tubes.

**Figure S3:** XRD pattern for granite samples MA‐N, 09Al105, MOB‐1 and GR8. Peak contributions are labelled with symbols for quartz, plagioclase, alkali feldspar, and muscovite. Full‐pattern summation was performed using the software powdR.

**Data S1:** Supporting Information.

## Data Availability

The data that support the findings of this study are available in the Supporting Information.
