# Supplementary material for: Experimental Evaluation and Optimisation of Sealed‐Tube Combustion Methods for Silicate Nitrogen Extraction
Source: Rapid Commun Mass Spectrom. 2026 Oct 14;41(1):e70193. doi: 10.1002/rcm.70193 (PMC13669138; doi:10.1002/rcm.70193)
Supplement: Supplementary file 1 [file RCM-41-e70193-s001.pdf]

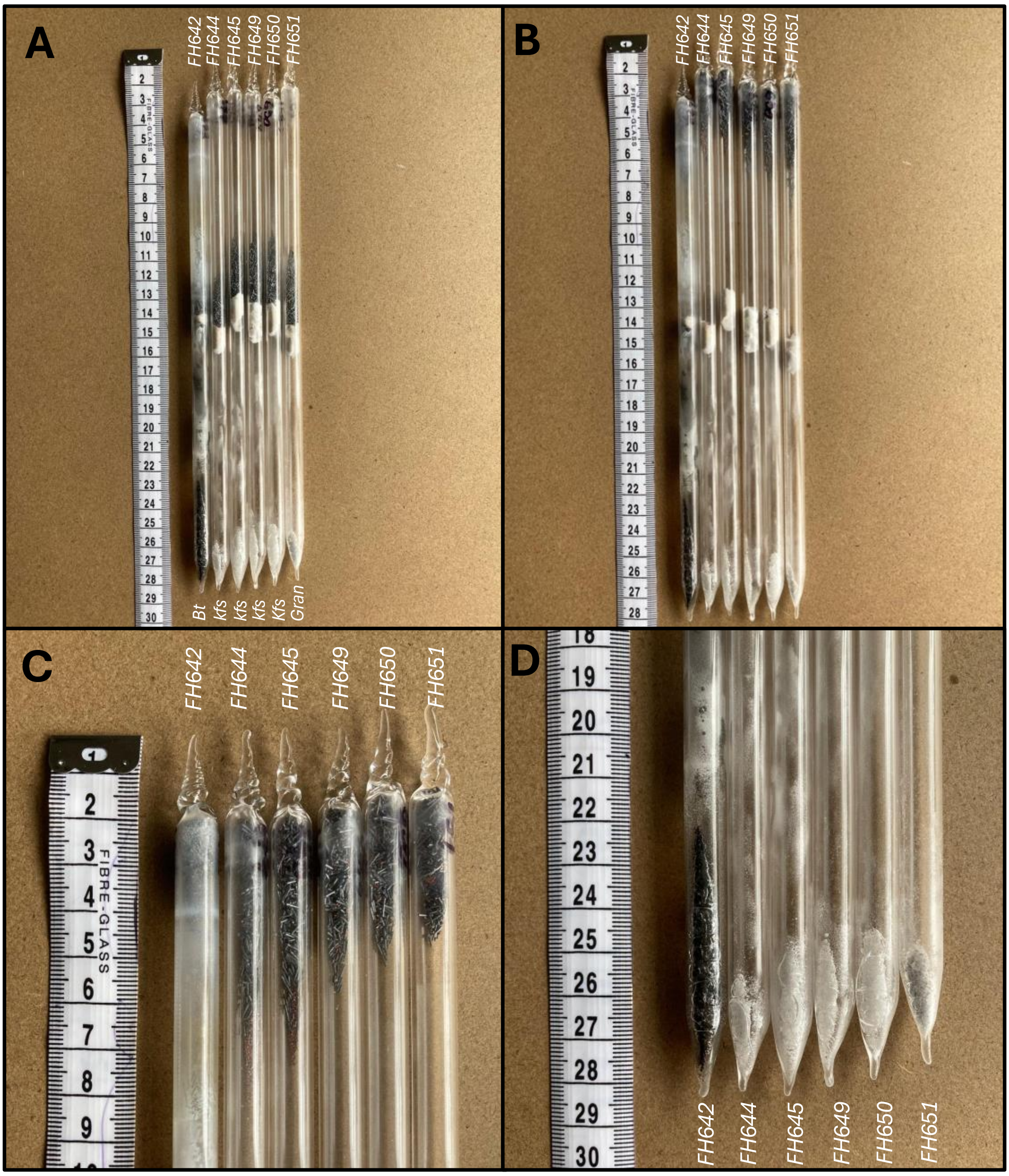

Supporting Information Figure 2. Granite (gran), alkali feldspar (ksf), and biotite bt) samples which melted, reacted with the glass tube, and caused the tube to crack. The vacuum seal was lost, and these samples were not measurable. These tubes were photographed to demonstrate that after the 1200 °C combustion step, the CuO and Cu pellets were still loose and did not melt. Therefore, CuO and Cu melting did not cause the tube to fail, and the failure was caused by a property of the sample powder itself. A) The CuO and Cu pellets fall towards the silica wool. B) The CuO and Cu pellets fall towards the tube end, away from the silica wool. Panels A and B together demonstrate that the pellets are still loose. C) Close-up of the CuO and Cu pellets. D) Close-up of the sample end of the tubes.
