# Supplementary material for: Experimental Evaluation and Optimisation of Sealed‐Tube Combustion Methods for Silicate Nitrogen Extraction
Source: Rapid Commun Mass Spectrom. 2026 Oct 14;41(1):e70193. doi: 10.1002/rcm.70193 (PMC13669138; doi:10.1002/rcm.70193)
Supplement: Supplementary file 2 [file RCM-41-e70193-s003.pdf]

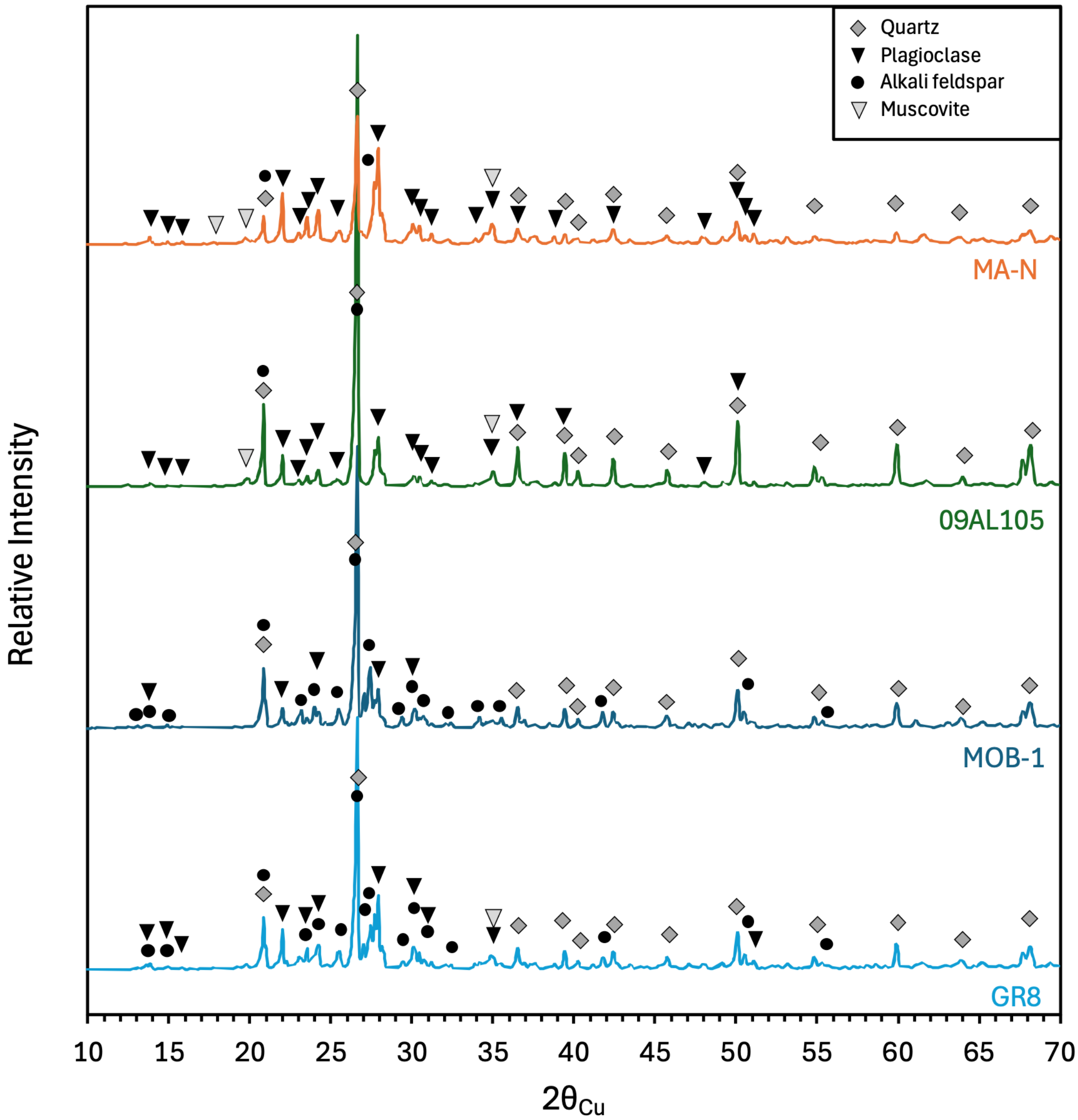

Supporting Information Figure 3. XRD pattern for granite samples MA-N, 09Al105, MOB-1 and GR8. Peak contributions are labelled with symbols for quartz, plagioclase, alkali feldspar, and muscovite. Full-pattern summation was performed using the software powdR.
