# Supplementary material for: Experimental Evaluation and Optimisation of Sealed‐Tube Combustion Methods for Silicate Nitrogen Extraction
Source: Rapid Commun Mass Spectrom. 2026 Oct 14;41(1):e70193. doi: 10.1002/rcm.70193 (PMC13669138; doi:10.1002/rcm.70193)
Supplement: Supplementary file 3 [file RCM-41-e70193-s004.pdf]

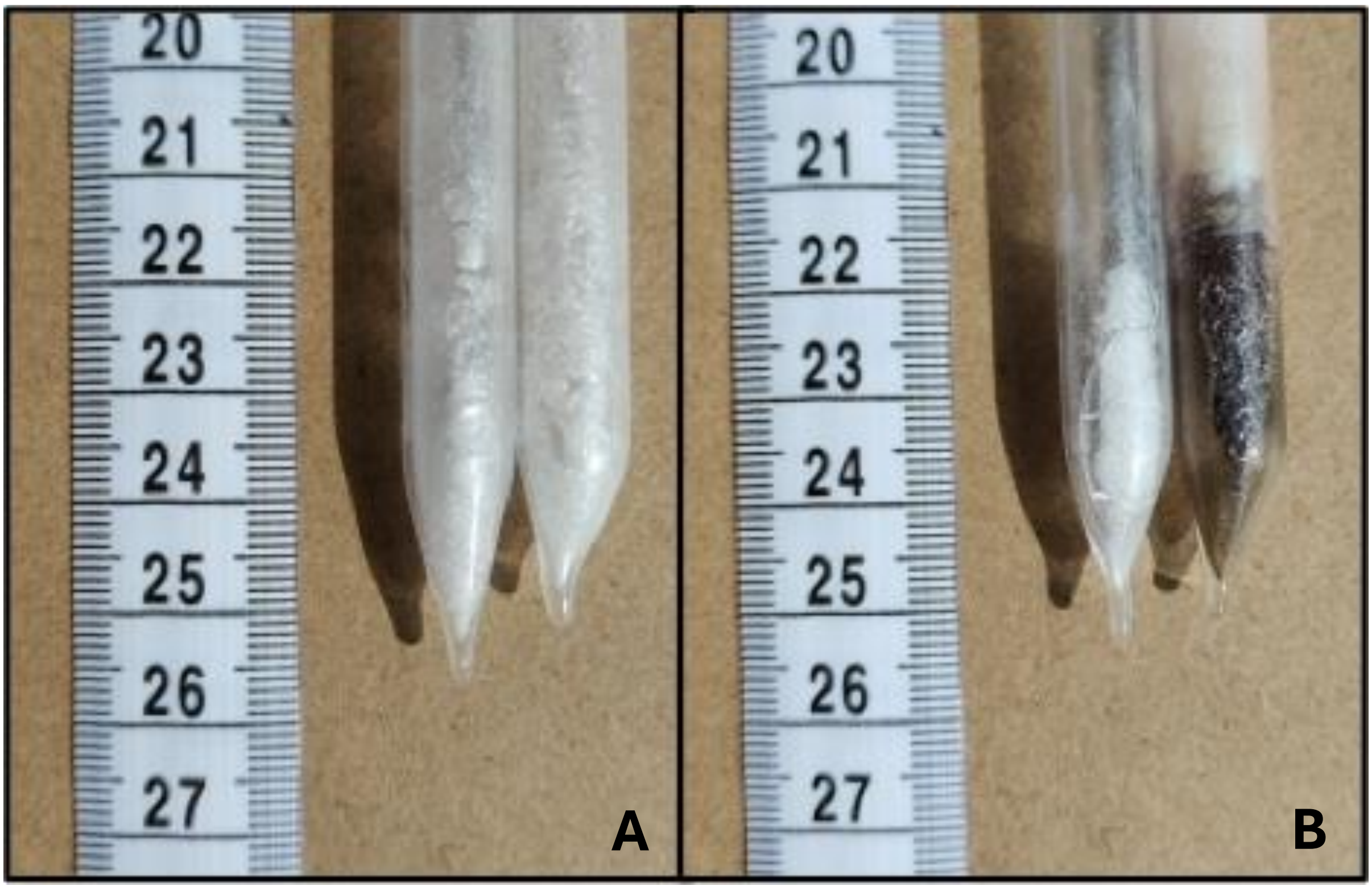

Supporting Information Figure 1. A) Two plagioclase samples, which melted and reacted with the silica glass tube, but the reaction was not so intense that it caused the tube to crack. These samples were measurable. B) Plagioclase (left) and biotite (right) samples, which melted and reacted with the glass tube; the reaction was so intense that it caused the tube to crack. These samples were not measurable.
